# Cultural drivers and health-seeking behaviours that impact on the transmission of pig-associated zoonoses in Lao People’s Democratic Republic

**DOI:** 10.1186/2049-9957-4-11

**Published:** 2015-03-02

**Authors:** Stephanie Burniston, Anna L Okello, Boualam Khamlome, Phouth Inthavong, Jeffrey Gilbert, Stuart D Blacksell, John Allen, Susan C Welburn

**Affiliations:** Division of Infection and Pathway Medicine, College of Medicine and Veterinary, Medicine, University of Edinburgh, 49 Little France Crescent, Edinburgh, EH16 4SB UK; CSIRO Animal Food and Health Sciences, Australian Animal Health Laboratory (AAHL), Regional Programme, 5 Portarlington Road, East Geelong, Victoria 3219 Australia; Department for Communicable Disease Control (DCDC), Ministry of Health, Thadeua Road, Vientiane, Lao PDR; Department of Livestock and Fisheries, Ministry of Agriculture and Forestry, Ban Sithan Nua, Luang Prabang Road, Sikhottabong District, Vientiane, 7042 Lao PDR; International Livestock Research Institute (ILRI), Asia Programme, Kabete, Naivasha Road, Nairobi, 30709-00100 Kenya; Mahidol-Oxford Tropical Medicine Research Unit, Faculty of Tropical Medicine, Mahidol University, 420/6 Rajvithee Road, 10400 Bangkok, Thailand; Centre for Tropical Medicine, Nuffield Department of Clinical Medicine, Churchill Hospital, Old Road, Headington, Oxford, OX3 7LJ UK

**Keywords:** Sociocultural drivers, Pig-associated zoonoses, Clinical syndromes, Health seeking behaviours, Brucellosis, Q-fever, Trichinellosis, Hepatitis E, Leptospirosis, Japanese encephalitis, *Streptococcus suis*, Taeniasis-cysticercosis

## Abstract

Pig rearing is an important income source in the Lao People’s Democratic Republic (PDR), with many smallholder farmers using traditional free-range pig production systems. Despite the potentially significant health risks posed by pig production regarding pig-associated zoonoses, information on the sociocultural drivers of these zoonoses is significantly lacking. This review summarises the existing sociocultural knowledge on eight pig-associated zoonoses suspected to be endemic in Southeast Asia: brucellosis, Q fever (*Coxiella burnetii*), trichinellosis, hepatitis E virus, leptospirosis, Japanese encephalitis, *Streptococcus suis* and *Taenia solium* taeniasis-cysticercosis. It summarises current knowledge on these diseases grouped according to their clinical manifestations in humans to highlight the propensity for underreporting. A literature search was conducted across multiple databases for publications from 1990 to the present day related to the eight pig-associated zoonoses and the risk and impact connected with them, with Lao PDR as a case study. Many of these pig-associated zoonoses have similar presentations and are often diagnosed as clinical syndromes. Misdiagnosis and underreporting are, therefore, substantial and emphasise the need for more robust diagnostics and appropriate surveillance systems. While some reports exist in other countries in the region, information is significantly lacking in Lao PDR with existing information coming mainly from the capital, Vientiane. The disease burden imposed by these zoonoses is not only characterised by morbidity and mortality, but directly impacts on livelihoods through income reduction and production losses, and indirectly through treatment costs and lost work opportunities. Other factors crucial to understanding and controlling these diseases are the influence of ethnicity and culture on food-consumption practices, pig rearing and slaughter practices, hygiene and sanitation, health-seeking behaviours and, therefore, risk factors for disease transmission. Published information on the knowledge, attitudes and beliefs of people regarding pig zoonoses and their risk factors is also extremely limited in Lao PDR and the broader Southeast Asian region. The need for more transdisciplinary research, using a One Health approach, in order to understand the underlining social determinants of health and their impacts on health-seeking behaviours, disease transmission and, ultimately, disease reporting, cannot be more emphasized.

**Electronic supplementary material:**

The online version of this article (doi:10.1186/2049-9957-4-11) contains supplementary material, which is available to authorized users.

## Multilingual abstracts

Please see Additional file 1 for translations of the abstract into the six official working languages of the United Nations.

## Introduction

The Lao People’s Democratic Republic (PDR) lies in Southeast Asia’s Mekong Region, sharing its borders with China, Thailand, Vietnam, Cambodia and Myanmar. Classified as a low-middle-income country, approximately 23.2% of people lived below the poverty line and with a life expectancy of 68 years in 2012 [1, 2]. Agriculture is vitally important to lesser-developed economies in the region; the majority of Lao PDR’s estimated 6.9 million people live in rural areas, with the agriculture sector employing 82% of the labour force in 2003 [1, 3]. According to the 2005 population census, the country’s 49 official ethnic groups consist of over 100 subgroups, all with distinctly different languages, customs and beliefs [4, 5].

Like many countries in the region, livestock production, particularly pig rearing, is an important ancillary income source in Lao PDR, with many smallholder farmers utilising traditional free-range pig production systems [6]. Whilst the livestock sector’s contribution to the country’s gross domestic product (GDP) from agriculture remains small at 14.3% [7], the societal contribution of livestock – especially within local economies and at the household level as a source of cash and social capital – remains vitally important. Pig production appears to be a particularly important poverty buffer in northern Lao PDR, where approximately 70% of households rear domestic pigs [8]. Despite pig operations being on a smaller scale compared with neighbouring countries, evidence shows that the number of pigs produced increased by 71% between 1998 and 2008, in addition to a 77% increase in the output of pork products [9, 10].

Pig production, however, poses a health risk to those relying on this commodity for income, with a number of pig-associated zoonotic diseases endemic throughout Southeast Asia. Besides the dual burdens on animal and human health, zoonotic diseases can impact the exportability of livestock and associated products. Despite this, little relevant information is available on the sociocultural drivers of pig-associated zoonoses in Lao PDR, or indeed, in the wider Southeast Asia region.

This review summarises the sociocultural knowledge on eight pig-associated zoonoses endemic to Southeast Asia, grouped according to their clinical manifestations in humans to highlight the propensity for underreporting: brucellosis, Q fever (*Coxiella burnetii)*, trichinellosis, hepatitis E virus, leptospirosis, Japanese encephalitis, *Streptococcus suis* and *Taenia solium* taeniasis-cysticercosis. Systematically examining the current knowledge regarding the risk and impact of pig-associated zoonoses in Lao PDR enables us to identify areas that require further research, including those in upstream value chain events such as slaughter and meat hygiene practices.

## Review

A literature review was conducted across multiple databases including PubMed, BIOSIS, CAB Direct, Web of Science, Journal Citation Reports (JCR), ScienceDirect, Social Science Research Network (SSRN), Google Scholar and ProQuest Sociology, using word search combinations of the eight zoonotic diseases and associated syndromes: ‘disease/syndrome AND Lao PDR’ and ‘zoonotic disease AND Lao PDR’, as well as studies related to sociocultural beliefs about health and health-seeking behaviours, hygiene and sanitation, food-consumption practices and pig slaughter practices in Lao PDR (including published references from these studies). Study titles and abstracts were screened, with full articles obtained and re-evaluated for inclusion/exclusion under the following criteria:

i. *Inclusion criteria:* Epidemiological studies, case reports, commentaries, reviews, letters, editorials, social research, conference proceedings and available grey literature published in English from 1990 to 2014.
ii. *Exclusion criteria:* Imported cases, genetic/molecular studies, development and evaluation of diagnostic and therapeutic techniques, diseases/syndromes of interest in animals other than pigs and articles in a languages other than English.

Based on the above criteria, a total of 430 abstracts were reviewed, with 142 full text reviews and 34 papers included in the final analysis. The results of the analysis are described below.

### I) Acute febrile illness and other ‘influenza-like’ symptoms: brucellosis, Q fever (*Coxiella burnetii*) and trichinellosis

Acute febrile illness, often accompanied by chills, headache and muscle pain, is a common manifestation of several infectious diseases endemic to Southeast Asia, many of which are zoonotic [11]. A summary of some of the pig-associated zoonoses suspected to be endemic in Southeast Asia that can cause these symptoms is illustrated in Table 1. The true burden of causative agents for acute febrile illness remains poorly understood in Lao PDR, with the majority (>50%) of published data from the region referring to Thailand, followed by Vietnam (27%) [12]. Whilst existing information on fever epidemiology and management in Lao PDR comes mainly from Vientiane, differences in the underlying aetiologies of fever – including co-infections – have been demonstrated in various geographical and ethnic regions [12–14].

**Table 1:** **Pig-associated zoonoses suspected to be endemic in Southeast (SE) Asia that can cause high fever, muscle pain and other influenza-like symptoms (acute febrile illness)**

Disease	Aetiological agent	Transmission route	Host animals in SE Asia	Estimated DALYs lost per year	Risk factors
Brucellosis	Bacterial (*Brucella* spp.)	Consumption of unpasteurised dairy and raw animal products; direct contact with infected animals/animal products; inhalation	Varies by region and strain – sheep, goats, cattle, buffalo, pigs	Unknown	Swine contact in high risk occupations; food preparation and consumption practices
Q-fever	Bacterial (*Coxiella* spp.)	Consumption of unpasteurised dairy and raw animal products; direct/indirect contact with infected animals/animal products; inhalation	Cattle, sheep, goats, cats, dogs, pigs, rodents, ticks, wildlife	Unknown	Swine contact in high risk occupations; food preparation and consumption practices
Trichinellosis/Trichinosis	Parasitic (*Trichinella spp.*)	Consumption of raw/undercooked pork products	Mainly pigs; also wild boar, cattle, sheep, dogs, cats, rodents, wildlife	Unknown	Pig husbandry; poor meat inspection; food preparation and consumption practices

a) Brucellosis: Brucellosis is a bacterial zoonosis with several animal reservoirs; *Brucella suis* being the strain carried by pigs. Presenting as a recurrent febrile illness with associated muscle and joint pain, with more serious consequences in 5% of patients [15], brucellosis represents a significant public health concern in developing contexts [16]. Although no reliable estimate of disability-adjusted life year (DALY) parameters currently exists for brucellosis, reduced transmission between animals can result in a significant number of DALYs averted [17]. Whilst no data currently exists on brucellosis prevalence in Lao PDR, the disease has been highlighted as a growing concern in neighbouring Thailand and China [18–20].

*B. suis* transmission occurs via consumption of raw animal products, or via direct contact with bodily fluids, tissues, aborted foetuses and aerosols [21, 22]. Although raw dairy products are not implicated in transmission in Lao PDR [23], the relatively common practice of raw meat, blood or offal consumption has been linked to infection in other countries, along with unregulated slaughter practices [22]. Pig raising practices are very important, with suggestions that rural populations – particularly smallholder farmers, butchers and animal health providers – are at a higher risk of infection than urban dwellers [24–27].

b) Q fever (*Coxiella burnetii*): A recognised public health concern in both developed and developing countries, *Coxiella burnetii,* or Q fever, outbreaks are frequently reported worldwide [28–31]. *C. burnetii* is an obligate intracellular bacterium able to remain highly virulent for long periods in the environment [32, 33]. The disease has a wide host range; it is detected in both domestic and wild animals, rodents and arthropods [28, 33–35]. The distribution of Q fever in Southeast Asia is not fully understood, and despite no published information available from Lao PDR in humans and pigs, it has been detected in cattle and buffalo in the northern part of the country [36]. Human cases have been detected in Thailand, Japan, China, South Korea and Malaysia [37–40].

Similar to brucellosis, the major clinical demonstration of Q fever is acute fever, with a potentially fatal vascular form developing in up to 5% of cases [32, 37]. Q fever is also a noteworthy cause of foetal morbidity and mortality [28]. Humans typically become infected via contact with infected animals or inhalation/ingestion of the bacteria from environmental contamination, often arising from infected birthing materials [32]. Pigs were implicated in several studies from the United States and the Netherlands [29–31]. The most common risk factor for smallholder pig farmers in Lao PDR is through slaughter, birth assistance and consumption of raw animal products [28, 37].

c) Trichinellosis*: Trichinella* is a nematode infection common to Southeast Asia, particularly in rural areas [41–45]. Three of the eight *Trichinella* nematode species have been documented in Southeast Asia to date – *T. spiralis*, *T. pseudospiralis* and *T. papuae* – all of which are associated with human disease [43–45]. *T. spiralis* is the species suspected to be endemic to Lao PDR, however, only three outbreaks have been documented to date [43, 46–50]. *T. papuae* is known to cause human trichinellosis in northern Thailand [51]. Though it has not yet been detected in Lao PDR, cross-border movement of animals between the two countries could facilitate the spread of this species into the latter country. Disease severity is influenced by the number of larvae ingested, with secondary effects including respiratory failure, myocarditis, encephalitis and acute adrenal gland failure resulting in death in some cases [42, 47, 49, 52].

*Trichinella* is transmitted through the consumption of raw or undercooked meat [48]. Weddings and other traditional ceremonies have been implicated in reported outbreaks in Lao PDR, China and Thailand [42, 43, 47], with raw pork the primary source of human infection [47, 49, 53]. Smallholder pig production practices including free roaming pigs that can access *Trichinella*-infected food scraps have been consistently linked to infections [47, 54–57].

### II) Acute jaundice syndrome: leptospirosis and hepatitis E virus

Acute jaundice is often defined as an acute onset of jaundice accompanied by severe illness, and has multiple and varied potential aetiologies. As with acute febrile illnesses, limited diagnostic capability makes definitive diagnosis near impossible. Therefore, the true burden of causative agents of acute jaundice is unknown. In the case of hepatitis as a cause for acute jaundice, the 2010 Global Burden of Disease (GBD) Study estimated that mortality due to hepatitis was 307,700 (ranging between 268,200 and 356,500) worldwide [58]. Estimated DALYs lost due to hepatitis was approximately 13,258,000 (11,364,000–15,855,00) or 192 (165–230) per 100,000 [59]. There is little information on the diverse infectious causes of jaundice and hepatitis in Southeast Asia. A summary of some of the pig-associated zoonoses that can cause these symptoms and are suspected to be endemic in the region is illustrated in Table 2. Certainly in the case of Lao PDR, very few studies have been published on the aetiology of jaundice and/or liver impairment, and those that exist are mainly out of the capital city, Vientiane [60, 61].

**Table 2:** **Pig-associated zoonoses suspected to be endemic in Southeast Asia that can cause acute jaundice and/or liver impairment**

Disease	Aetiological agent	Transmission route	Host animals in SE Asia	Estimated DALYs lost per year	Risk factors
Hepatitis E	Viral (hepatitis E virus)	Direct contact with urine, blood and other secretions from infected animals; environmental contamination	Varies by region – rodents, cattle, pigs, dogs	3, 715 000 DALYs (between 1,552,000 and 7,470,000)	Outdoor recreation; swine contact in high risk occupations; poor hygiene and sanitation; flooding (wet season)
Leptospirosis	Bacterial (*Leptospira spp.)*	Direct contact with urine, blood and other secretions of infected animals; consumption of raw/undercooked pork products; environmental contamination (rivers and streams)	Mainly pigs; also deer, horse, cattle, sheep, goats, cats, dogs, rodents and macaques	Unknown	Swine contact in high risk occupations; food preparation and consumption practices; flooding (wet season); single water source; poor hygiene and sanitation; use of pig manure as fertiliser for vegetable gardens

a) Leptospirosis: Leptospirosis is a bacterial zoonotic infection with a worldwide distribution. The causative agent is the spirochaete *Leptospira interrogans*, divided into 24 serogroups (>200 serovars). A reliable estimate of DALY parameters is not yet available for leptospirosis, although the disease is increasingly recognised as an important cause of acute jaundice and febrile illness in Southeast Asia [62–64]. Globally, an estimated 300,000–500,000 human cases of severe disease occur each year [65]. Countries that presumed to have high incidence rates (>10 per 100,000 population) in the Asia-Pacific region are Lao PDR, Bangladesh, Cambodia, Nepal, Thailand, Vietnam and others [66]. No official data exists for Lao PDR, Vietnam and Cambodia, but some studies have confirmed that the disease is endemic in these countries. Leptospirosis outbreaks are usually associated with tropical wet season flooding [66, 67], consistent with findings in Lao PDR where a higher number of hospital presentations occur during the wet season [14]. Poor sanitation is a major contributor to environmental contamination, for instance, through open sewers and waste disposal outlets.

Humans are accidental hosts, with farmers and butchers at increased risk [66, 68]. Leptospires from infected animal urine enter the body through skin cuts/abrasions or mucous membranes, either through direct contact from slaughter or farming practices, or via contaminated water or soil [69, 70]. Disease severity varies, with acute hepatitis and haemorrhagic pneumonitis suspected in 5–10% of cases [68]. Mortality rates range between 5–40%, with pulmonary failure being a major contributor to poor prognosis [66].

Reservoir hosts can vary according to the serovar and geographic region, with domestic and wild pigs implicated in transmission [68]. Whilst leptospirosis has been reported in pigs in Thailand and Vietnam [71–74], no data exists from Lao PDR. One study from Vietnam suggested an association between pig rearing practices and level of risk, particularly in wet areas where free-range pigs have an increased chance of exposure to leptospires in soil and water [74, 75]. Another risk for pig infection is exposure to feed or water supplies contaminated with infected urine from rodents or other pigs [74].

b) Hepatitis E virus (HEV): The global epidemiology and distribution of the hepatitis E virus has changed significantly in recent years, manifesting as large outbreaks of acute hepatitis in regions with poor sanitation and hygiene [76, 77]. Virus genotypes 1 and 2 generally cause large waterborne outbreaks in humans, while genotypes 3 and 4 are primarily zoonotic [78, 79]. The latest GBD Study estimated that 3,715,000 DALYs (between 1,552,000 and 7,470,000) are lost globally from acute hepatitis E [59], with approximately 56,600 deaths occurring worldwide in 2010 [58]. The disease is generally reported in rural areas in Southeast Asia, with a high case fatality rate (CFR), especially in pregnant women (10–24%) [80]. Some evidence of human HEV infection exists in Lao PDR [61, 80, 81], however, the extent and impact is not fully understood compared with other causes such as hepatitis A [60].

Studies have shown that swine-related occupations incur a greater risk of human HEV infection [78, 82]. An important risk factor in Lao PDR is the consumption of raw or undercooked pork [83, 84]. Direct contact with infected pigs or ingestion of pig faeces as a result of poor village-level sanitation have also been suggested as sources of infection [78, 85]. Sporadic cases tend to be food borne, while waterborne outbreaks are common during the rainy season [86]. In Lao PDR, further research is needed to ascertain whether seasonality has an influence on virus transmission, particularly of the zoonotic genotypes.

### III) Epilepsy and other neurological conditions: Japanese encephalitis virus, *Taenia solium*cysticercosis, *Streptococcus suis*

Central nervous system (CNS) infections are associated with high morbidity and mortality, often with long-term neurological and psychiatric sequelae. The 2010 GBD Study estimated that 7,141,000 (6,148,000–8,274,000) and 9,563,000 (8,108,000–10,858,000) DALYs are lost globally due to infectious causes of encephalitis and meningitis^a^, respectively [59]. Central nervous systems infections are also the leading cause of epilepsy in tropical developing countries, with estimated prevalence ranging from 10% to 40%; high compared to the mean global prevalence of 8% [87]. Of these cases, 80–94% were reportedly not adequately treated [87]. Rapid and early diagnosis is key to favourable patient outcomes; hence knowledge of the aetiological agents of CNS infections is critical in order to guide empirical therapy. However in Lao PDR, adequate diagnostic facilities are generally confined to the capital Vientiane, and the common practice of antibiotic administration prior to diagnostic testing impedes definitive diagnosis, especially in rural areas [88]. Apart from a single study using a polymerase chain reaction (PCR) for diagnosis of bacterial meningitis [88], no publications could be found on the underlying aetiologies of CNS infections in Lao PDR. A summary of some of the pig-associated zoonoses suspected to be endemic in Southeast Asia that can cause these symptoms is also illustrated in Table 3.

**Table 3:** **Pig-associated zoonoses suspected to be endemic in Southeast Asia that can cause epilepsy and other neurological conditions**

Disease	Aetiological agent	Transmission route	Host animals in SE Asia	Estimated DALYs lost per year	Risk factors
Japanese encephalitis (JE)	Viral (JE virus)	Vector borne: *Culex tritaeniorhynchus*	Waterfowl (ducks, herons, egrets), pigs, horses	709,000 for JE; 7, 141 000 (6,148,000–8,274,000) for encephalitis	Vector population and wet season; rice agriculture production and its proximity of the household; pig husbandry systems
*Taenia solium* taeniasis-cysticercosis	Parasitic (*T. solium*)	Consumption of raw/undercooked pork or vegetable products; ingestion of viable cysts from infected pork, faecal-oral route (taenia eggs)	Pigs	503,000 (379,000–663,000)	Food preparation and consumption practices; poor hygiene and sanitation; pig husbandry systems; use of human faeces as fertiliser for vegetable gardens; poor meat inspection
*Streptococcus suis*	Bacterial (*S. suis*)	Consumption of raw/undercooked pork products; direct contact with carrier or infected pigs/pig products	Pigs mainly; also wild boars, horses, dogs, cats	Unknown for *S. Suis*; 9, 563 000 (8,108,000–10,858,000) for meningitis^i^	Food preparation and consumption practices; swine contact in high risk occupations; slaughter practices

^i^Meningitis other than pneumococcal meningitis, *H. influenzae* type B meningitis and meningococcal infection.

a) Japanese encephalitis virus (JE): Japanese encephalitis is the primary cause of viral encephalitis in Asia [89]. An estimate of the JE global burden was 709,000 DALYs in 2002, however, the true burden is suspected to be much higher [90, 91]. It has an estimated global annual incidence of approximately 35–50,000 human cases, and an annual mortality of 10–15,000 deaths [92, 93]. Of the survivors, 30–50% have severe neurological and psychiatric sequelae [91, 94]. Its contribution to epilepsy is noteworthy, given that up to 65% of JE patients suffer from acute seizures and 13% have chronic epilepsy [95, 96]. Serological evidence of the virus exists in both pigs and humans throughout Lao PDR [6, 14, 92, 97–99], but cross-reaction with other flaviviruses makes definitive diagnosis difficult [100].

Significant in rural settings, the mosquito vector breeds in rice fields, with a zoonotic transmission cycle maintained by pigs and ardeid wading birds [101]. Studies have shown a link between the proximity to irrigated rice fields and JE transmission, where the onset of the monsoon rain season sees prolific growth in the mosquito population [89, 102]. Pigs are the primary amplifying hosts, given their high natural infection rate and a propensity for mosquitoes to feed on them, thus acting as maintenance hosts in endemic areas [101]. During times of peak transmission, the close proximity of pigs and humans drives epidemic transmission to humans in a phenomenon sometimes referred to as the ‘spillover effect’. JE transmission is therefore multifactorial, driven by interactions between pigs, humans and the rural environment [89].

b) *Taenia solium* cysticercosis: *Taenia solium* cysticercosis results from the ingestion of tapeworm eggs from contaminated environments, with encystment of the larval form in the CNS resulting in neurocysticercosis (NCC). Neurocysticercosis is a major cause of human epilepsy across Asia, with wide prevalence variations between different regions and socioeconomic groups [103]. Worldwide mortality due to NCC is estimated at about 50,000 [104], with significant social and economic consequences for survivors as a result of chronic disability [105]. The latest GBD Study estimated that 503,000 (379,000–663,000) DALYs are lost globally due to cysticercosis [59].

Reported prevalences of NCC in Lao PDR are limited, although there have been several historical case reports [106–110]. While *T. solium* taeniasis has been confirmed in Lao PDR, its relationship to epilepsy has not been fully elucidated [109, 111]. However, it has been suggested that NCC is a likely cause of epilepsy in areas with high human taeniasis prevalence [96]; Conlan et al. [109] found a relatively high prevalence of cysticercosis in young children that corresponded with high taeniasis burdens in the same age group. Local customs regarding pork consumption and latrine usage play a major role in the persistence and spread of taeniasis and cysticercosis in endemic regions. This was evident in a recent cross-sectional survey conducted in northern Lao PDR where a high prevalence of taeniasis re-infection was observed where open defecation and uncooked pork consumption were common practices in a considerable proportion of the study population [109]. Poverty and religious beliefs have been shown to influence desire to discard cyst-infected meat or consume it uncooked, particularly during sacrificial ceremonies [112].

c) *Streptococcus suis*: *Streptococcus suis* is a common bacterial infection that naturally inhabits the respiratory, genital and alimentary tracts of pigs [113]. High pig densities and frequent consumption of raw or undercooked pork means over 50% of global *S. suis* cases occur in Asia [114–116]. The lack of understanding of the epidemiological situation in Southeast Asia is a significant concern, particularly since antimicrobial drug resistance has emerged in Vietnam [113, 117].

Recognised as a leading cause of bacterial meningitis and septicaemia in humans, *S. suis* manifests as septicaemia, endocarditis and acute meningitis [113, 117–121]. Survivors are often left with neurological sequelae, with mortality reported in 2.6–20% of cases, usually as a result of septic shock [113, 119, 120, 122–124].

*S. suis* is commonly found in Southeast Asian communities that consume raw or undercooked pork, including blood and viscera. Whilst no prevalence data exists on *S. suis* infections in Lao PDR, food-consumption practices in parts of the country are similar to Vietnam and Thailand, where the disease has been reported [94, 119, 120, 125]. Alcohol consumption has also been suggested as a potential risk factor [119, 124]. Similarly to leptospirosis and JE*, S. suis* seems to be common during the wet season, with some suggestion that weather-induced stress precipitates the infection in pigs, leading to human spillover [119, 124, 126]. Those in close or direct contact with pigs or unprocessed pig products, particularly during slaughter, are at particularly high risk [118, 119, 126].

### Common practices that can significantly impact transmission of these diseases

#### Pig rearing, slaughter and pork consumption

Like many countries across the world, pigs play an important socioeconomic role in Lao PDR, central to traditional ceremonies and income generation for smallholder farmers [23]. Because of poverty, lack of market access and relatively high risks of pig loss due to disease outbreaks, existing pig production systems in much of the country are traditional and with low investment. Understanding pig production, slaughter and consumption practices is critical to identifying and minimising the risks to producers, traders, butchers and others working closely with pigs who have a high risk of exposure to these zoonoses. Additionally, identifying and understanding supply chain networks and cross-border trading points is important to the development of sustainable and robust disease surveillance and monitoring systems.

Choudhury et al. [46] highlighted the importance of understanding the sociocultural beliefs and practices of the Lao people, contextualised within cross-border trade risks and the resulting economic burdens of zoonotic disease in pigs. For example, the frequent cross-border trade with Vietnam in the north of Lao PDR, coupled with a cultural propensity for raw pork consumption, highlights the potential for the circulation of diseases such as *T. solium, Trichinella* and *S. suis* between the two countries. Ethnicity, culture and religion play an important role in influencing the type of food eaten and how it is prepared. The consumption of raw pork (‘*lap-mou*’), blood and offal products poses a risk of transmission of a variety of zoonotic diseases, including *Trichinella*, *T. solium* and HEV. Tran et al. [87] found raw meat consumption was practiced by about 30% of epilepsy patients in central Lao PDR, indicating *T. solium* NCC may be the primary cause. An understanding of the different food preparation and consumption rituals amongst the numerous and diverse ethnic groups in Lao PDR could help develop culturally acceptable interventions and impact on the zoonotic disease burden [46, 87, 109, 111].

The rise in demand for pork and pork products can overwhelm national public health systems, leading to an increased risk of exposure to zoonotic pathogens through contact or consumption of meat from informal market chains [118]. Poor meat inspection is a documented risk factor for transmission of these zoonoses, particularly in poor rural areas, and slaughterhouse data within Lao PDR is notably limited [8, 50, 127]. Improper slaughter can result in contamination of pork meat, thus increasing the risk of infection to workers and consumers of pig-associated zoonoses such as brucellosis, Q fever, leptospirosis, HEV and *S. suis*. Occupational exposure has been implicated in deaths from *S. suis* in Thailand, Vietnam and China [117, 120, 121]. Despite this, access to protective equipment when handling raw pork products is limited in many rural communities and slaughter points in Lao PDR (personal observations A L Okello, S D Blacksell). Whilst changing consumption practices may not be possible, the impact of improved meat inspection regulations, hygiene practices at pork processing facilities, and community education programmes related to food safety, pig management and pork handling practices could have significant impact on the level of disease transmission from pigs to humans.

### Water, hygiene and sanitation

Leptospirosis, HEV, *Taenia solium* and Japanese encephalitis are all implicated in rural areas suffering from poor sanitation and wet season flooding, with the 2005 National Population and Housing Census indicating that less than 20% and approximately 50% of people in northern Lao PDR had access to clean water and sanitation, respectively [5]. The mountainous northern part of the country in particular suffers from a lack of social and economic development given the limited transportation of commodities, communication and other exchanges [128].

Kawaguchi et al. [129] found an association between leptospirosis and a history of recent flooding; interestingly the risk was higher in women. Similar to HEV, waterborne exposure to leptospires is greater in rural areas, given the reliance on natural water sources for drinking and bathing purposes. Recent studies in Lao PDR have also found cultural influences on attitudes to open defecation, and that ownership of a latrine does not always denote its use [87, 130]. In addition, gender differences in latrine use and handwashing practices have translated into differences in worm burdens between males and females in parts of the country [130]. As Bardosh et al. [112] describes, the social acceptability of open defecation, especially among males, lessens the desire for latrines.

### Sociocultural perspectives

Published information on the knowledge, attitudes and beliefs of people regarding pig zoonoses and their risk factors for transmission is extremely limited, both in Lao PDR and the broader Southeast Asia region. Vertical control programmes involving chemotherapy and health education are often implemented without comprehension and consideration of community beliefs and perceptions, resulting in high re-infection rates [104, 130]. In Lao PDR, studies have found that some ethnicities have an aversion to taking therapeutics if not deemed ‘sick’, impacting the efficacy of prevention programmes such as mass drug administration (MDA) for parasite control [112, 131]. Common misconceptions about the underlying causes of disease symptoms also exist in Lao PDR, including amongst healthcare workers. Epilepsy associated with *T. solium* is a good example, with common beliefs including that the condition is contagious, transmitted through routine activities such as sharing meals or has supernatural origins [131–133]. Where such beliefs are circulating, control and advocacy of these zoonoses will be even more difficult.

The recruitment and compliance of study participants in Lao PDR also varies depending on which ethnic group they belong to [109, 111, 112]. For instance, some ethnic groups have an aversion to giving blood and are embarrassed by giving faecal samples. This can impact the likelihood of their participation in prevalence surveys, and their understanding of risk and propensity to seek treatment, ultimately leading to underreporting. The benefits of transdisciplinary research approaches in order to understand the underlining sociocultural determinants of health, and to thereby develop culturally appropriate interventions, cannot be emphasised enough.

### Healthcare access and health-seeking behaviours in Lao PDR

Health-related outcomes in Lao PDR remain among the poorest in Southeast Asia [4]. Much of the country is rural, with people scattered in mountainous, hard-to-reach areas, thereby impacting accessibility to basic healthcare facilities [2, 4]. Acute diarrhoea, respiratory infections, parasites and vaccine-preventable diseases remain major causes of morbidity and mortality, further compromised by overarching development issues such as inadequate sanitation, poor nutrition and under-resourced healthcare facilities [2]. Complicating disease surveillance and control is the limited diagnostic capacity in Lao PDR, particularly outside Vientiane. Provincial healthcare workers rely on symptomatic treatments for commonly presenting syndromes such as fever, neurological signs and jaundice. The lack of definitive diagnoses further impacts on the accuracy of prevalence data.

Ethnicity, culture and religion play important roles in influencing health-seeking behaviours in Lao PDR. Government health expenditure remains relatively low, with recent figures at less than 1% of the country’s GDP [134]. With a strong vertical approach to health service delivery and heavy reliance on development partner funding, Lao PDR has a government-run public system that is significantly underutilised, particularly in rural areas. Where development agency subsidies do not exist, health expenditure is covered by out-of-pocket payments, placing many households at further risk of poverty if illness occurs. As ability to pay and distance to a healthcare provider are often major barriers, self-medication is common practice in Lao PDR [135]. For this reason, the role of ethnicity, culture and religion in influencing health-seeking behaviours is important to understand. For example, health is commonly associated with spiritual balance as opposed to factors surrounding nutrition, hygiene and healthcare practices [136, 137]. Some, including healthcare workers, also believe that traditional medicine offers the possibility of cure where modern medicine cannot [138]. The general beliefs and perceptions surrounding health, a high illiteracy rate and difficulties in access to health services further aggravate the already low level of healthcare utilisation [136]. Traditional medicine receives strong support from both citizens and the government of Lao PDR. Reasons for its use include perceived efficacy, cultural acceptance, minimal side effects, accessibility and lower cost in comparison to modern medicine [4, 138]. Lao PDR’s government has expressed commitment to health system strengthening, as stated in the goals of the Ministry of Health’s 7^th^ Five-Year National Health Sector Development Plan (2011–2015).

## Conclusion

As a considerable proportion of the population in Lao PDR relies on smallholder pig production for its economic survival and sociocultural role, pig-associated zoonoses can greatly impact livelihoods both directly through production losses, and indirectly through costs incurred through treatment and lost work opportunities. There is a strong need for collaborative research, more robust diagnostic technologies and a greater understanding of the sociocultural determinants of health within the various ethnicities of Lao PDR and the wider Southeast Asian region more generally. A drive to better integrate transdisciplinary human and animal health approaches under the One Health approach would not only increase the cost effectiveness of disease prevention and control, but also improve the efficiency of disease surveillance and response, thus strengthening veterinary and human health systems as a whole. By highlighting the potential for misdiagnosis and underreporting of many pig-associated zoonoses, this review contributes valuable evidence to suggest that the current World Health Organization (WHO) list of neglected zoonotic diseases (NZDs) could be expanded to include several zoonoses pertaining to the important role of smallholder pig production in Asia, for example *Streptococcus suis,* Japanese encephalitis and hepatitis E virus.

## Endnotes

^a^Causes of meningitis other than pneumococcal meningitis, *H. influenzae* type B meningitis and meningococcal infection.

## Authors’ information

SB is a doctoral candidate at the University of Edinburgh, researching human and animal health systems in Lao PDR. ALO is a postdoctoral researcher at the University of Edinburgh currently managing an Australian government-funded pig zoonoses project in Lao PDR. BK is a medical doctor at the Lao Ministry of Health Department for Communicable Disease Control (DCDC). PI is the Deputy Director of the National Animal Health Laboratory (NAHL) in the Lao PDR Department of Livestock and Fisheries. JG is both a medical doctor and veterinarian with significant experience in managing zoonoses control and research programmes in the Asia region. SDB is a senior scientist at the Mahidol-Oxford Tropical Medicine Research Unit in Bangkok, with extensive research experience in pig zoonoses in Lao PDR. JA is regional programme manager at the Australian Animal Health Laboratory (AAHL), a managing agent for the One Health Smallholder Pig Systems Project (SPSP), which conducted the research. SCW is the Director of the Global Health Academy and Assistant Principal for Global Health at the University of Edinburgh.

## Electronic supplementary material

Additional file 1: **Multilingual abstracts in the six official working languages of the United Nations.** (PDF 296 KB)

## Abbreviations

CFR: Case fatality rate
CNS: Central nervous system
DALY: Disability-adjusted life years
GBD: Global burden of disease
HEV: Hepatitis E virus
JE: Japanese encephalitis
NCC: Neurocysticercosis
NZD: Neglected zoonotic disease
PDR: People’s democratic republic
WHO: World health organization.
