# Supplementary material for: Cultural drivers and health-seeking behaviours that impact on the transmission of pig-associated zoonoses in Lao People’s Democratic Republic
Source: Infect Dis Poverty. 2015 Mar 2;4:11. doi: 10.1186/2049-9957-4-11 (PMC4430026; doi:10.1186/2049-9957-4-11)
Supplement: Supplementary file 1 — Additional file 1: Multilingual abstracts in the six official working languages of the United Nations. (PDF 296 KB) [file 40249_2014_100_MOESM1_ESM.pdf]

Translation of the abstract into the six official working languages of the United Nations

**الدوافع الثقافية وسلوكيات التماس العناية بالصحة التي تؤثر على انتقال الأمراض حيوانية المنشأ المرافقة للخنازير بين سكان جمهورية لاو الديمقراطية الشعبية**

ستيفاني بيرنستون، أنا ل. أوكيلو، بوالام خملوم، فوث إنثافونغ، جيفري جيلبرت، ستوارت د. بلاكسيل، جون ألين، سوزان س. ولبرن

**الملخص**

تعتبر تربية الخنازير مصدرا هاما للدخل في جمهورية لاو الديمقراطية الشعبية، حيث يستخدم العديد من صغار المزارعين نظم إنتاج الخنازير التقليدية المجانية. وعلى الرغم من المخاطر الصحية الكبيرة المحتملة التي يجلبها إنتاج الخنازير فيما يتعلق بالأمراض الحيوانية المنشأ المرافقة للخنزير، فالمعلومات الخاصة بالدوافع الاجتماعية والثقافية لهذه الأمراض الحيوانية المنشأ ناقصة بشدة. في هذه المراجعة موجز عن المعرفة الاجتماعية والثقافية الحالية المتعلقة بثمانية أمراض حيوانية المنشأ مرافقة للخنزير يشتبه في كونها أمراض متوطنة في جنوب شرق آسيا: داء البروسيلات، حمى Q (الكوكسيلة البورنيتية)، داء الشعرينات، فيروس التهاب الكبد E، داء اللولبية النحيفة والتهاب الدماغ الياباني، العقدية الخنزيرية داء الشريطيات - داء الكيسات المذنبة بالشريطية الوحيدة. وهو يلخص المعرفة الحالية بهذه الأمراض المجمّعة وفقا لمظاهرها السريرية لدى البشر لتسليط الضوء على الميل لعدم الإبلاغ عن حدوثها. وأجري بحث في أدبيات الإصابات عبر عدد من قواعد البيانات الخاصة بالدوريات المنشورة منذ عام 1990 وحتى يومنا هذا ذات الصلة بالأمراض الثمانية الحيوانية المنشأ المرافقة للخنزير، والمخاطر والآثار المرتبطة عليها، مع اعتبار جمهورية لاو الديمقراطية الشعبية حالة مدروسة. يبدو العديد من هذه الأمراض الحيوانية المنشأ المرافقة للخنزير بشكل متماثل، وغالبا ما تُشخّص كمتلازمات سريرية. لذا فإن الخطأ في التشخيص وعدم الإبلاغ مرتفعان ويؤكدان الحاجة لإجراءات تشخيصية أقوى و لأنظمة مراقبة مناسبة. ورغم وجود بعض التقارير في بلدان أخرى من المنطقة، فالمعلومات غير متوفرة بشكل واضح في جمهورية لاو الديمقراطية الشعبية، وترد المعلومات الموجودة من العاصمة فينتيان بشكل رئيسي. لا يتصف عبء الأمراض التي تفرضها هذه الأمراض الحيوانية المنشأ بالأمراض المشاركة والوفيات فحسب، ولكن يؤثر مباشرة على سبل العيش عن طريق إنقاص الدخل وخسائر الإنتاج، وبشكل غير مباشر عن طريق تكاليف العلاج وخسارة فرص العمل. العوامل الأخرى الحاسمة لفهم هذه الأمراض والسيطرة عليها هي التأثيرات الإثنية والثقافية على ممارسات استهلاك الغذاء، وطرق تربية الخنازير وذبحها، والنظافة والصرف الصحي، وسلوكيات التماس العناية بالصحة، وبالتالي، عوامل الخطر المرافقة لانتقال المرض. المعلومات المنشورة عن المعرفة والسلوكيات ومعتقدات الناس المتعلقة بأمراض الخنزير الحيوانية المنشأ وعوامل الخطر المرافقة لها محدودة جدا هي الأخرى في جمهورية لاو الديمقراطية الشعبية ومنطقة جنوب شرق آسيا الأوسع. الحاجة إلى إجراء بحوث بين كافة التخصصات، باستخدام نهج صحي واحد، من أجل التركيز على المحددات الاجتماعية للصحة وتأثيرها على سلوكيات التماس الرعاية الصحية، وانتقال المرض، والإبلاغ عن الأمراض بالنتيجة، لا يمكن التعبير عنه أكثر من ذلك.

Translated from English version into Arabic by Lina SM, through

TRANSLATORS
WITHOUT BORDERS

**文化驱动和就医行为对猪相关人兽共患病在老挝人民民主共和国传播的影响**

Stephanie Burniston, Anna L Okello, Boualam Khamlome, Phouth Inthavong, Jeffrey Gilbert, Stuart D Blacksell, John Allen, Susan C Welburn

**摘要**

生猪饲养在老挝人民民主共和国（PDR）是一项重要的收入来源，许多小型农户在生猪饲养中使用传统的自由放养生产体系。尽管在生猪生产中有潜在重大的人兽共患病的健康风险，这些疾病的社会文化驱动的相关信息仍明显缺乏。本综述总结了 8 种可能在东南亚流行的猪相关人兽共患病的现有社会文化知识，分

别是：布氏杆菌病、Q 热（立克次体）、旋毛虫病、戊型肝炎病毒、乙型脑炎、钩端螺旋体病、猪链球菌病和猪带绦虫病囊虫病。本综述总结上述疾病的社会文化的现有知识，依据其临床表现对其分组，以突出这类疾病漏报或低估的倾向。自多个数据库将老挝 1990 年至今的 8 种猪相关人兽共患病作为个案调查进行文献检索，调查其风险和相关影响。这些猪相关人兽共患病例多有相似的表现，常被诊断为临床综合征。产生了大量误诊和漏报，因此强调需要更强大的诊断和适当的监测系统。一些报告存在于该地区的其他国家，信息明显缺乏。老挝现有资料主要来自首都万象。这些人兽共患病的疾病负担的不仅是发病率和死亡率，还有因收入减少和生产损失而对生计有直接影响，以及因治疗费用和失去工作机会等间接影响。其他对理解和控制这些疾病非常重要的因素是对食物消费行为、生猪饲养和屠宰行为、保健与卫生、就医行为和疾病传播的相关危险因素。文献报道老挝和更广泛的东南亚地区人民对猪相关人兽共患病和其危险因素的知识、态度和信念显得极为有限。因此，强调需进行更多跨学科研究并实施同一个健康理念来理解健康的社会决定因素与这些因素对就医行为、疾病传播和疾病报告的影响。

Translated from English version into Chinese by Zheng Qi, through

CHINESE CENTER FOR DISEASE CONTROL AND PREVENTION
NATIONAL INSTITUTE OF PARASITIC DISEASES

**Facteurs culturels et comportements favorisant la santé influant sur la transmission des zoonoses porcines en République démocratique populaire Lao**

Stephanie Burniston, Anna L Okello, Boualam Khamlome, Phouth Inthavong, Jeffrey Gilbert, Stuart D Blacksell, John Allen, Susan C Welburn

**Résumé**

L'élevage porcin est une importante source de revenus en République démocratique populaire (RDP) Lao, où de nombreux petits exploitants pratiquent l'élevage traditionnel en liberté. Malgré les risques sanitaires potentiellement importants de cet élevage, liés aux zoonoses porcines, il n'y a globalement pas d'informations sur les facteurs socioculturels de ces zoonoses. Cette revue résume les connaissances socioculturelles existantes sur huit zoonoses porcines suspectées d'être endémiques en Asie du Sud-est : brucellose, fièvre Q (*Coxiella burnetii*), trichinellose, hépatite E, leptospirose, encéphalite japonaise, *Streptococcus suis* et téniase-cysticercose à *Taenia solium*. Elle résume les connaissances actuelles sur ces maladies, regroupées selon leurs manifestations cliniques chez l'homme, de manière à mettre en évidence une tendance au sous-signalement. Une recherche a été menée dans plusieurs bases de données afin de remonter les publications datées de 1990 à ce jour en relation avec ces huit zoonoses porcines et leurs risques et impacts, dans le cadre d'une étude de cas sur la RDP Lao. Beaucoup de ces zoonoses porcines se manifestent par des symptômes similaires et sont souvent diagnostiquées comme des syndromes cliniques. Les erreurs de diagnostic et sous-signalements sont donc fréquents, ce qui met en évidence la nécessité de méthodes de diagnostic plus fiables et de systèmes de surveillance appropriés. Bien qu'il y existe quelques signalements dans d'autres pays de la région, les informations manquent dans l'ensemble en RDP Lao et les informations existantes proviennent surtout de la capitale, Vientiane. Le fardeau imposé par ces zoonoses n'est pas seulement lié à la morbidité et à la mortalité ; il grève aussi les moyens de subsistance, que ce soit directement en réduisant les revenus et en détruisant la production, ou indirectement par le coût des traitements et les emplois perdus. D'autres facteurs essentiels pour comprendre et endiguer ces maladies comprennent l'influence de l'appartenance ethnique et de la culture dans les pratiques de consommation des aliments, les pratiques d'élevage

et d’abattage des porcs, l’hygiène et l’assainissement, les comportements favorables à la santé et, partant, les facteurs de risque de transmission de la maladie. Les informations publiées sur les connaissances, les attitudes et les croyances des populations à propos des zoonoses porcines et de leurs facteurs de risques sont extrêmement limitées en RDP Lao et, au-delà, dans toute l’Asie du Sud-est. On ne saurait trop insister sur la nécessité de nouvelles recherches transdisciplinaires utilisant une approche « One Health/Une seule santé », pour comprendre les déterminants sociaux sous-jacents de la santé et leur influence sur les comportements favorables à la santé, la transmission de ces maladies et, en dernier recours, leur signalement.

Translated from English version into French by Suzanne Assenat, through

**Культурные стимулы и тенденции активного обращения за медицинской помощью, которые оказывают воздействие на распространение передаваемых свиньями зоонозов в Лаосской Народно-Демократической Республике**

Стефани Бернистон, Анна Л. Окелло, Буалам Хамлом, Фут Интавонг, Джеффри Гилберт, Стюарт Д. Блэкселл, Джон Аллен, Сюзан Си Уэлберн

**Краткое описание**

Разведение свиней – это важный источник дохода в Лаосской Народно-Демократической Республике (ЛНДР), где многие фермеры до сих пор используют традиционные неограниченные системы разведения свиней. Несмотря на потенциально высокие риски для здоровья, которые представляет собой свиноводство в отношении передаваемых свиньями зоонозов, информации о социокультурных стимулах таких зоонозов катастрофически не хватает. Настоящий обзор обобщает существующие на данный момент социокультурные знания о восьми передаваемых свиньями зоонозах, случаи которых, вероятно, были зафиксированы в Юго-восточной Африке: бруцеллез, ку-лихорадка (*Coxiella burnetii*), трихинеллез, вирус гепатита Е, лептоспироз, японский энцефалит, *Streptococcus suis* и *Taenia solium* тениоз-цистицеркоз. В нем представлены актуальные знания об этих заболеваниях, классифицированные согласно их клиническим проявлениям у человека с тем, чтобы обратить внимание на склонность к намеренному занижению сведений. Был проведен поиск по литературным источникам в рамках разнообразных баз данных, включая публикации с 1990 года по настоящий момент по теме восьми передающихся свиньями зоонозов, а также обусловленных ими рисков и воздействий, при этом ситуационным исследованием являлась ЛНДР. Большинство передаваемых свиньями зоонозов имеют схожие проявления и зачастую диагностируются как клинические синдромы. Таким образом, постановка неправильного диагноза и занижение сведений встречаются повсеместно, что подчеркивает необходимость проведения более тщательной диагностики и использования соответствующих систем мониторинга. Хотя в некоторых странах данного региона составлены соответствующие отчеты, в ЛНДР информации не хватает, при этом основные данные приходят из столицы – Вьентьян. Данные зоонозы характеризуются не только высоким уровнем заболеваемости и смертности, но и прямым воздействием на качество жизни за счет понижения дохода и производственных потерь, и косвенным воздействием за счет расходов на лечение и упущенных рабочих

возможностей. Другие факторы, играющие важную роль при понимании и контроле данных заболеваний, включают влияние этнической составляющей и культуры на практику потребления пищи, разведение и забой свиней, гигиену и санитарию, обращение за медицинской помощью, а, значит, факторы риска при передаче заболевания. Опубликованная информация об имеющихся знаниях, отношения и убеждения людей в отношении зоонозов и их факторов риска также носят чрезвычайно ограниченный характер в ЛНДР и по всему региону Юго-восточной Азии. Нельзя не отметить необходимость в проведении дополнительных междисциплинарных исследований с использованием подхода One Health для того, чтобы понять скрытые социальные факторы состояния здоровья и их воздействие на тенденции активного обращения за медицинской помощью, передачу заболеваний и, в конечном счете, на сообщение сведений о заболевании.

Translated from English version into Russian by Irina Zayonchkovskaya, through

TRANSLATORS
WITHOUT BORDERS

**Factores culturales y comportamientos que aspiran a mejorar la salud que influyen en la transmisión de las zoonosis porcinas en la República Democrática Popular Lao**

Stephanie Burniston, Anna L. Okello, Boualam Khamlome, Phouth Inthavong, Jeffrey Gilbert, Stuart D. Blacksell, John Allen, Susan C. Welburn

**Resumen**

La crianza de cerdos es una importante fuente de ingresos en la República Democrática Popular Lao (RDP) y muchos ganaderos pequeños utilizan sistemas de producción de cerdos al aire libre tradicionales. A pesar de los importantes riesgos de salud que conlleva la producción de cerdos en cuanto a zoonosis porcinas, hay una enorme falta de información sobre los factores socioculturales de este tipo de zoonosis. En esta reseña se resume la información con la que se cuenta sobre los factores socioculturales de ocho zoonosis porcinas que se sospecha serían endémicas en el sudeste asiático: la brucelosis, la fiebre Q (*Coxiella burnetii*), la triquinosis, el virus de la hepatitis E, la leptospirosis, la encefalitis japonesa, el *Streptococcus suis* y la teniasis/cisticercosis por *Taenia solium*. Para resumir la información con la que se cuenta actualmente, se agruparon estas enfermedades según sus manifestaciones clínicas en seres humanos para destacar la propensión a que muchos casos no se den a conocer. Se ha llevado a cabo una búsqueda bibliográfica en varias bases de datos entre publicaciones desde 1990 a la fecha relacionadas con las ocho zoonosis porcinas y los riesgos e impactos asociados con ellas, tomándose la RDP Lao como población del estudio de caso. Muchas de estas zoonosis porcinas se presenta de manera similar y suelen diagnosticarse como síndromes clínicos. Los diagnósticos erróneos y los casos que no se dan a conocer son, por lo tanto, considerables y ponen de relieve la necesidad de contar con diagnósticos más sólidos y sistemas de vigilancia adecuados. Si bien hay algunos informes en otros países de la región, existe una gran falta de información en la RDP Lao, en donde la información que sí hay proviene principalmente de la capital, Vientián. La carga de enfermedad que imponen estas zoonosis no solo se caracteriza por su morbosidad y mortalidad, sino que afectan directamente el sustento de las personas ya que reducen los ingresos y generan pérdidas de producción, e indirectamente a través de los costos de los tratamientos y la pérdida de oportunidades laborales. Otros factores

cruciales para comprender y controlar estas enfermedades son la influencia de las prácticas de consumo de alimentos, crianza y matanza de cerdos propias de cada etnia y cultura, así como sus hábitos de higiene, sanidad y comportamientos que aspiran a mejorar la salud, y por consiguiente, los factores de riesgo de transmisión de enfermedades. La información publicada sobre el conocimiento, las actitudes y las creencias de la gente con respecto a las zoonosis porcinas así como sus factores de riesgo también es extremadamente limitada en la RDP Lao y la región del sudeste asiático en general. La necesidad de realizar investigaciones más transdisciplinarias, con un enfoque One Health, para poder comprender los factores sociales subyacentes que determinan la salud y sus efectos en los comportamientos que aspiran a mejorar la salud, la transmisión de enfermedades y, en última instancia, el informe de los casos de enfermedades no puede ser más evidente.

Translated from English version into Spanish by María Emilia Meini, through

TRANSLATORS
WITHOUT BORDERS
